# Immunity to Increasing Condition Numbers of Linear Superiorization versus Linear Programming

**Published:** 2024-07-26

**Authors:** Jan Schröder, Yair Censor, Philipp Süss, Karl-Heinz Küfer

**Affiliations:** 1 Optimization Department, Fraunhofer - ITWM, Kaiserslautern 67663, Germany; 2 Department of Mathematics, University of Haifa, Mt. Carmel, Haifa 3498838, Israel

**Keywords:** Condition number, ill-posed problem, Linear Programming, Linear Superiorization, bounded perturbations, feasibility seeking, Scipy, Gurobi

## Abstract

Given a family of linear constraints and a linear objective function one can consider whether to apply a Linear Programming (LP) algorithm or use a Linear Superiorization (LinSup) algorithm on this data. In the LP methodology one aims at finding a point that fulfills the constraints and has the minimal value of the objective function over these constraints. The Linear Superiorization approach considers the same data as linear programming problems but instead of attempting to solve those with linear optimization methods it employs perturbation resilient feasibility-seeking algorithms and steers them toward feasible points with reduced (not necessarily minimal) objective function values. Previous studies compared LP and LinSup in terms of their respective outputs and the resources they use. In this paper we investigate these two approaches in terms of their sensitivity to condition numbers of the system of linear constraints. Condition numbers are a measure for the impact of deviations in the input data on the output of a problem and, in particular, they describe the factor of error propagation when given wrong or erroneous data. Therefore, the ability of LP and LinSup to cope with increased condition numbers, thus with ill-posed problems, is an important matter to consider which was not studied until now. We investigate experimentally the advantages and disadvantages of both LP and LinSup on examplary problems of linear programming with multiple condition numbers and different problem dimensions.

## 1. Introduction

In this paper we investigate and discuss the behavior of the linear superiorization (LinSup) methodology for various different condition numbers and compare it to the behavior of multiple linear optimization solvers. We set up differently conditioned linear optimization problems in a manner that keeps them structurally similar in order to keep them comparable to each other. Then we solve these problems with the introduced superiorization methodology and with established implementations of the simplex algorithm and an interior point method. We compare the necessary runtimes in problems with multiple dimensions and discuss the observed effects. We show experimentally that superiorization is rather robust in comparison to scipy’s simplex implementations observing both faster runtimes and better objective function values.

### 1.1. Context and previous work

The superiorization methodology (SM) is a relatively new method that aims to improve an existing iterative algorithm by interlacing into it perturbation steps ([10]). In the context of optimization problems, this concept can be applied by interlacing into a feasibility-seeking algorithm (for example a projection method) perturbations of negative gradient steps that reduce the objective function values. Since its development, the SM has successfully been applied in various practical applications such as intensity-modulated radio therapy ([3]), image reconstruction ([11]) and telecommunication networks ([17]). In the particular context of linear optimization problems (LPs), there is a vast literature for both the feasibility problem (for example the Agmon-Motzkin-Schönberg algorithm [1, 24],[14, algorithm 5.4.1]) and the optimization problem (e. g., simplex algorithm, ellipsoid method [25]). Furthermore, for the LinSup case, the “guarantee problem of the superiorization method” has been answered positively. This problem is the question whether superiorization can actually converge to a feasbile point with a lower or equal objective function value than the point of the unsuperiorized version of the same feasibility-seeking algorithm. In [13] the authors employ the principle of concentration of measure to show the result with high probability. A similar conclusion for the nonlinear case is yet to be found.

This paper aims to compare the superiorization methodology with optimization algorithms for LPs in an organized, reproducible and fair manner for problems of varying difficulty, indicated by the problems’ dimensions and their condition numbers. Accordingly, our work is an extension of the results in [9], where a superiorization method for linear problems (called LinSup) was compared to the simplex method. We augment these results by varying the condition numbers across multiple problem instances. The condition number has been known to have a large impact on the performance of certain methods and often leads to the failure of an algorithm ([28]). As many real-world problems have an inherently high condition number, immunity against ill-conditioning of problems is a desirable property for any algorithm. Often it is enough to have a rough understanding of the order of magnitude of the condition number and there are efficient algorithms for its estimation ([15],[22]). There is a vast literature on preconditioning of matrix problems, which has been reviewed in [5].

### 1.2. Outline

We give a brief overview of the superiorization method and of condition numbers in Sections 2.1 and 2.2, respectively. We set up multiple linear optimization problems with varying condition numbers in Section 3.1 and run the superiorization method and several LP algorithms on them. For details of our implementation of the superiorization method see Section 3.2. In Section 4 we present the results, followed by a discussion of remaining challenges and future work in Section 5.

## 2. Preliminaries

### 2.1. The superiorization methodology

The Superiorization Methodology (SM) developed from the investigation of feasibility-seeking models of some important real-world problems such as image reconstruction from projections and radiation therapy treatment planning. Feasibility-seeking algorithms, mainly projection methods, generate iterative sequences that (under reasonable conditions) converge to a point in the feasible set. Their main advantage is that they perform projections onto the individual sets whose intersection is the feasible set and not directly onto the feasible set and the underlying situation is that such projections onto the individual sets are more manageable.

When one wishes to find feasible points with a reduced, not necessarily minimized, value of an imposed objective function then the SM comes into play. The principle of the SM is to interlace into the iterates of a feasibility-seeking iterative process perturbations that will steer the iterates toward superior (meaning smaller or equal) objective function values without losing the overall convergence of the perturbed iterates to a feasible point. To this end “bounded perturbations” are used.

How all this is done is rigorously described in earlier papers on the SM, consult, e.g., [10] for a recent review, read also [20]. A key feature of the SM is that it does not aim for a constrained optimal function value, but is content with settling for a feasible point with reduced objective function value – reduced in comparison to the objective function value of a feasible point that would be reached by the same feasibility-seeking algorithm without perturbations. For many applications this is sufficient, in particular, whenever the introduction of an objective function is only a secondary goal. Fulfillment of the constraints, in this context, is considered by the modeler of the real-world problem to be much more important, see, e.g., [10, 11, 3].

Many papers on the SM are cited in [8] which is a Webpage dedicated to superiorization and perturbation resilience of algorithms that contains a continuously updated bibliography on the subject. This Webpage is a source for the wealth of work done in this field to date, including two special issues of journals [12] and [18] dedicated to research of the SM. Recent work includes [7, 19, 21, 23, 29].

### 2.2. Condition numbers

The relative condition number is a measure for the impact of deviations in the input data on the output data of a problem. In particular, it describes the factor of error propagation when given wrong or erroneous data. Let the function $f:{\mathbb{R}}^{n}\to {\mathbb{R}}^{m}$ represent some mathematical problem and let $x\in {\mathbb{R}}^{n}$ be its input, where ${\mathbb{R}}^{n}$ stands for the Euclidean n-dimensional space. Denote with $\tilde{x}\in {\mathbb{R}}^{n}$ the disturbed input data. Then the relative condition number of the problem at the point x is defined as (see [31, equation (12.4)])

(1) $$\kappa_{\text{rel}}:=\underset{\tilde{x}\to x}{\text{lim sup}}\frac{\left\|f(\tilde{x})-f(x)\right\|}{\left\|\tilde{x}-x\right\|}\frac{\left\|x\right\|}{\left\|f(x)\right\|},$$

as long as f(x)≠0. Otherwise, it is $\kappa_{\text{rel}}=\infty$. In particular, the condition number is independent of a chosen numerical algorithm for solving the problem f, but the algorithms convergence speed may depend on the magnitude of the condition number (see [28, Section 4]). In the following we are interested in the condition number of matrices. When f(x)=Ax describes the problem of matrix multiplication, where $A\in {\mathbb{R}}^{m\times n}$ and with $\left\|\cdot \right\|={\left\|\cdot \right\|}_{2}$, the above formula becomes

(2) $$\kappa_{\text{rel}}=\underset{\tilde{x}\to x}{\text{lim sup}}\frac{\left\|A(\tilde{x}-x)\right\|}{\left\|\tilde{x}-x\right\|}\frac{\left\|x\right\|}{\left\|Ax\right\|}.$$

As f is differentiable and writing $\tilde{x}-x=hv$, for some unit vector v and $h=\left\|\tilde{x}-x\right\|$ we get

(3) $$\kappa_{\text{rel}}=\underset{h\to 0}{\lim}\frac{\left\|A(x+hv-x)\right\|}{h}\frac{\left\|x\right\|}{\left\|Ax\right\|}=\left\|A\right\|\frac{\left\|x\right\|}{\left\|Ax\right\|}\leq \left\|A\right\|\left\|A^{-1}\right\|.$$

The term on the right is called the condition number of the matrix A (see [31, equation (12.15)]), where $A^{-1}$ denotes the inverse or, if A is non-square, the pseudo-inverse of A:

(4) $$\kappa (A)=\left\|A\right\|\left\|A^{-1}\right\|=\frac{\sigma_{\max}}{\sigma_{\min}},$$

where $\sigma_{\max}$ and $\sigma_{\min}$ are the maximal and minimal singular values of A, respectively. The condition number plays a significant role in the analysis of numerical problems and is subject to extensive studies in the literature ([16], [31], [15], [28]). Several methods exist to improve high condition numbers of ill-conditioned problems (these are the, so-called, pre-conditioning methods, see e. g., [5]) in order to increase the accuracy of calculated solutions. This is often necessary because many real-world applications give rise to condition numbers of significant magnitude. This is the key motivation for the investigation in this paper.

## 3. Problem Setup and Implementation Details

### 3.1. The problem formulation

We consider linear problems of the form

(5) $$\begin{array}{c} \underset{x\in {\mathbb{R}}^{n}}{\min}\langle c,x\rangle \\ \text{s}.\text{t}.Ax\leq b\\ \;\ell \leq x\leq u, \end{array}$$

where $A\in {\mathbb{R}}^{m\times n},b\in {\mathbb{R}}^{m},c,\ell ,u\in {\mathbb{R}}^{n}$. Write A=UΣV via the singular value decomposition (cf. [6]) with semi-orthogonal matrices U and V and diagonal matrix $\text{Σ}=\text{diag}\left(\sigma_{1},\ldots ,\sigma_{q}\right)$ of singular values. Without loss of generality, let $\sigma_{1}\geq \ldots \geq \sigma_{q}\geq 0$. The condition number κ of A is (see [16])

(6) $$\kappa (A)=\frac{\sigma_{\max}}{\sigma_{\min}}=\frac{\sigma_{1}}{\sigma_{q}}.$$

We want to construct a sequence of matrices $A_{\kappa }$ of a specified condition number κ in such a way that they remain structurally similar to each other. To this end we reverse the singular value decomposition, that is, we create exactly one pair of U and V which contains the structure of the problem and construct, for different values of κ, diagonal matrices ${\text{Σ}}_{\kappa }$, which impose the condition number of the problem. Then, we calculate $A_{\kappa }:=U{\text{Σ}}_{\kappa }V$. As any matrix has a singular value decomposition, this makes it possible to define any matrix via this approach too. In our construction we focus on matrices of full rank, because otherwise one can remove rows or columns until full rank is achieved. In order to construct Σ, setup q=min(m,n)=rank(A) singular values as

(7) $$\sigma_{i}=\frac{t}{z_{i}}+\frac{1-t}{s}$$

where $t=\frac{\kappa -1}{q-1}$, s=10 and $z_{i}=\frac{si}{q}$. Note that this choice is somewhat arbitrary. We chose this setup of the $\sigma_{i}$, because in real-world applications the singular values often behave approximately proportional to $\frac{1}{i}$ (instead of linear which seems like an obvious first choice for our problem). The parameter s is used to control the magnitude of the singular values since in this model we always have $\sigma_{q}=\frac{1}{s}$. Due to the choice of t it is easy to see that

(8) $$\frac{\sigma_{1}}{\sigma_{q}}=\frac{\sigma_{\max}}{\sigma_{\min}}=\kappa$$

as desired. For U and V choose random semi-orthogonal matrices and set $A=U\cdot \text{diag}\left(\sigma_{1},\ldots ,\sigma_{q}\right)$ · V. Furthermore, set b=A𝟙+𝟙, u=−ℓ=100·𝟙 and randomly choose C. This choice of parameters implies the feasibility of x=𝟙. We set up this problem for multiple dimensions, ranging from 80×100 to 2000 × 2500 (cf. [9]).

## 3.2. The superiorization algorithm

We aim to apply separately linear programming and the superiorization methodology for the data $A={\left(a^{i}\right)}_{i=1}^{m},b,c,\ell ,u$ that appears in (5). In the SM we chose for the feasibility-seeking algorithm the projection method of Agmon Motzkin and Schönberg (AMS) with overshoot $0<r:=10^{-3}$ as the “basic algorithm”. This algorithm cyclically projects onto the individual half-spaces $\langle a^{i},x\rangle \leq b_{i}$ via (as defined in [27, p. 411])

(9) $$A_{i}(x):=\left\{ \begin{array}{l} x-\frac{\langle a^{i},x\rangle -b_{i}+r\left\|a^{i}\right\|}{{\left\|a^{i}\right\|}^{2}}a^{i},\text{if}\langle a^{i},x\rangle >b_{i}\\ x\text{, otherwise} \end{array}\right.$$

and a full sweep through all half-spaces is done by the algorithmic operator A, which is a composition of the individual projections

(10) $$A:=A_{m}\circ \cdots \circ A_{1}.$$

The parameter r describes, how far into the half-space the projection maps (i. e., beyond the bounding hyperplane). For the direction vectors in the perturbations used in the SM we chose the normalized negative gradient of the objective function in (5), which is constant throughout and equals $-\frac{c}{\left\|c\right\|}$. For the step-sizes $\eta_{k}$ we take an exponentially decreasing null sequence ${\left(10\cdot 0.99^{n}\right)}_{n\in \mathbb{N}}$ with resets every $\tau_{\text{reset}}=20$ iterations as described in [2, p. 6]. The starting step-size $\eta_{0}=10$ is decreased by the kernel α=0.99 in each iteration, unless there is a reset. In that case, set $\eta_{k}=\eta_{0}\alpha^{\rho }$, where ρ is the number of previous resets during this run.

In other words, the k-th iteration consists of a gradient step $-\eta_{k}\frac{c}{\left\|c\right\|}$ followed by a cyclic sweep of projections onto the half-spaces via A as given in (9) and (10). This process was repeated, until the iterate $x^{k}$ was feasible up to a tolerance of $\epsilon =10^{-8}$ and the relative change $\frac{\left\|x^{k}-x^{k-1}\right\|}{\left\|x^{k-1}\right\|}$ from the previous iterate was negligible, i. e., became smaller than ϵ. Algorithm 21 contains the pseudocode for the described process. All runs were initialized at the all-ones vector $x^{0}=𝟙$. The parameters ϵ, α, $\eta_{0}$, r, $\tau_{\text{reset}}$ can be adjusted for individual preferences or a particular problem.

**Algorithm 1:** The superiorization algorithm

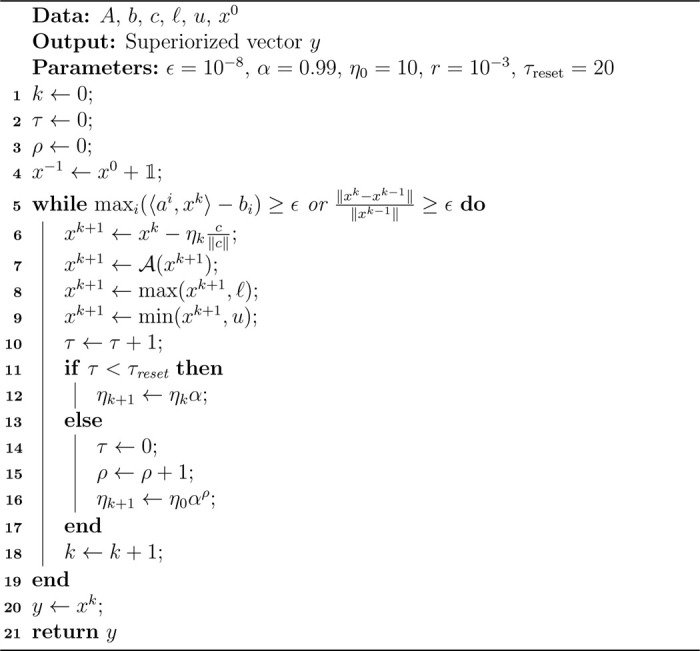

### 3.3. The LP algorithms

We compare the superiorization method with the following LP algorithms:

1. scipy.simplex
2. scipy.revised simplex
3. scipy.interior-point
4. gurobi.primal simplex.

Scipy is a library for scientific computing in the programming language Python. It is freely available at www.scipy.org. Its optimization suite *scipy.optimize* contains multiple implementations of common optimization algorithms like the SQP method, the dogleg method or the conjugate gradient method. Since we are working with the data of an LP, we employed specialized LP methods only, in particular the simplex and revised simplex method as well as the interior-point method of scipy’s *linprog* function. Gurobi is a commercial state-of-the-art solver for linear and nonlinear, continuous and (mixed) integer problems. It is available at www.gurobi.com and offers a wide range of customization, including multiple algorithms, globalization strategies, preconditioning, etc. For our experiments we turned off Gurobi’s automated choice of algorithm and instead forced it to use its implementation of the primal simplex to ensure consistency of the output data.

## 4. Experimental Results of the Comparative Investigation

Our main finding is, that, based on our computational experiments, the superiorization methodology is quite robust with respect to increasing condition numbers. In particular, the runtime remains relatively small when compared to the scipy implementations of the simplex algorithm. We conjecture that this is due to the fact, that our superiorization implementation never considers the full problem at once, but performs individual projections onto the half-spaces instead. This comes at the cost of sacrificing feasibility during early iterations. Furthermore, we conjecture, that the bounded perturbation resilience of the basic algorithm may play a role in absorbing errors that occur during computation, which would normally be amplified by the condition number.

**Figure 1:**
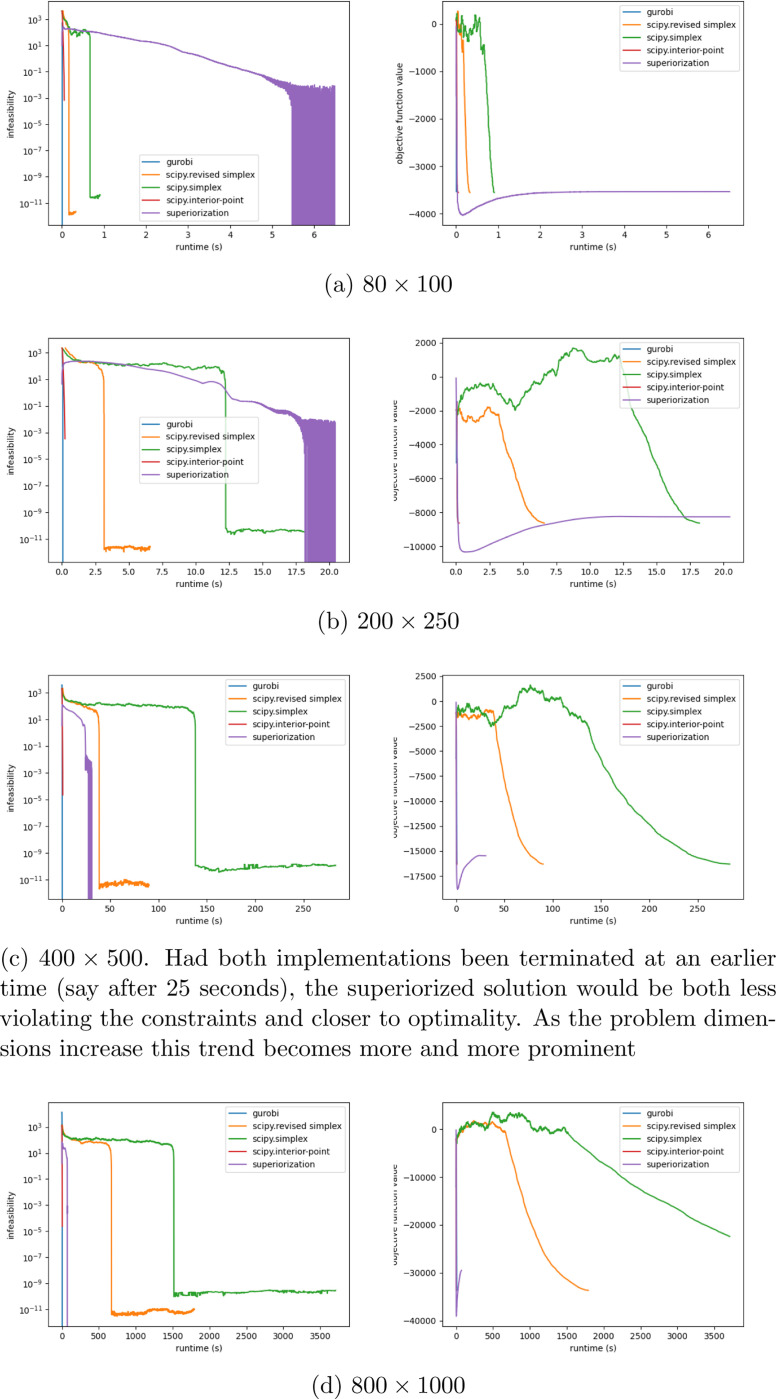
Figures 1a-1d show the behavior of the different algorithms on problems with increasing dimension for fixed condition number κ=1000. On the left we see the maximum violation of the constraints $\text{ma}{\text{x}}_{i}\left(\langle a^{i},x\rangle -b_{i}\right)$ plotted against the runtime. On the right we have the corresponding objective function values 〈c,x〉 plotted against the runtime.

Depending on the rate at which the step-sizes of the perturbations converge to zero, this would mean that superiorization, in general, may be less affected by high condition numbers, than other current algorithms. A secondary finding is, that superiorization can produce better results than the scipy simplex implementations, when both are terminated at a certain time, that is before the usual termination criterion is met, cf. Figure 1c. Furthermore, we see that superiorization reaches its termination criterion considerably faster than scipy in higher dimensions.

The trend is clear: While in problems with smaller dimensions the simplex algorithms are considerably faster, with increasing problem dimensions the scipy implementations take much longer to terminate. We also see a clear difference in the algorithms styles: Simplex aims to become feasible first and then starts to improve the objective function. Superiorization, on the other hand, reduces objective function values in its initial iterations because then the step-sizes of the perturbations are still large and only as iterations proceed the effect of feasibility-seeking becomes stronger.

Finally, it should be noted that scipy’s revised simplex performs better than scipy’s standard simplex method. This can be attributed to the internal LU decomposition of the basic matrices, leading to better numerical stability as described in [4].

We observe that in our experiments the interior point method and the Gurobi implementation outperformed the other algorithms by a lot. For the interior point method this matched our expectations and can be explained in the following way: The condition number of a matrix can be interpreted as a measure of how linearly dependent its rows or columns are. A well-conditioned matrix (i.e., κ=1) only has a single singular value and will be semi-orthogonal, whereas an ill-conditioned matrix (κ “large”) will have “almost” linearly dependent entries. Consequently, the half-spaces $\left\{x\in {\mathbb{R}}^{n}:\langle a^{i},x\rangle \leq b_{i}\right\}$ will be almost parallel and the resulting polyhedron will consist of many facets and vertices. A basic simplex implementation, which moves from vertex to vertex, will consequently face a long runtime. The interior point method, on the other hand, is not dependent on the vertices. It will take its path through the interior of the polyhedron regardless of its boundary.

The Gurobi implementation is based on observations of the condition number. In that sense, our experiments played into one of Gurobis strongest suits. As one of the biggest commercial state of the art solvers we did not expect to outperform Gurobi with our implementation, but rather used it as a benchmark to compare with the other algorithms.

## 5. Conclusions

In this paper we experimentally discussed the superiorization method and constrained optimization on a set of examplary linear problems with varying condition numbers and problem sizes with the aim of investigating and comparing their immunity to increasing condition numbers. Our experimental results are promising for the observed problem sizes, but more work needs to be done for larger instances to verify that the trend that we observed continues.

The superiorization method and constrained optimization use the same input data which consists of a family of constraints obtained from the modeling process along with a user-chosen objective function. But the two approaches aim at different end-points of their iterative processes. The easy accessibility of the superiorization methodology allows for quick implementations with the advance knowledge that the aim is not to reach a constrained optimum. At the same time the SM may compute its solutions at a lower runtime (in the case of scipy’s simplex and revised simplex).

As superiorization is a relatively new concept, we expect that, with further tuning of its parameters it will continue to find a place as a computational model and tool in situations in which users do not wish to invest efforts in seeking a constrained optimal point but rather wish to find a feasible point which is “superior” in the sense of having a smaller or equal objective function value than that of a feasible point reached by the same feasibility-seeking algorithm.

Another interesting point is that, as is well-known, interior point methods reach their performance limits for ill-conditioned nonlinear problems (e. g., in intensity-modulated radio therapy). It would be interesting to compare the superiorization methodology in a nonlinear setting, with a different basic feasibility-seeking algorithm, to the interior point method to see if superiorization can contribute to solving these problems faster.

## Figures and Tables

**Figure 2:**
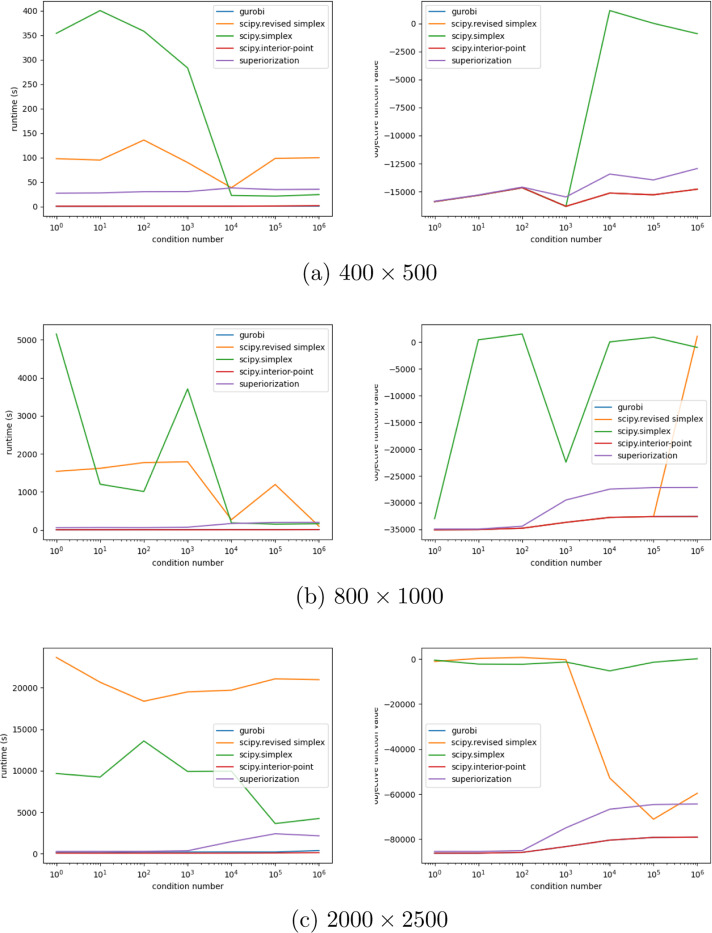
Figures 2a-2c demonstrate well the robustness with respect to increasing condition numbers. On the left we see the runtimes of each algorithm, plotted against the condition numbers, on the right we see the objective function value at termination, plotted against the condition numbers. Notice the severely suboptimal objective function value of scipy’s simplex for high condition numbers. This explains the low runtime as the algorithm realizes that these problems are hard and quickly “surrenders”. This trend continues in Figures 2b and 2c and the revised simplex too starts to reach its limits. Superiorization on the other hand proves to be quite stable in terms of its runtime with regards to increasing condition numbers.
